# Supervised vs. adversarially pre-trained pseudo-label generators for semi-supervised breast cancer patch classification

**DOI:** 10.3389/frai.2026.1899268

**Published:** 2026-08-31

**Authors:** Tomiris Zhaksylyk, Aruzhan Imasheva, Beibit Abdikenov, Birzhan Ayanbayev

**Affiliations:** Science and Innovation Center “Artificial Intelligence, ” Astana IT University, Astana, Kazakhstan

**Keywords:** adversarial training, breast cancer, contrastive learning, digital pathology, histopathology, pseudo-labeling, semi-supervised learning, whole-slide image

## Abstract

Automated breast cancer classification from whole-slide images (WSIs) is hampered by the scarcity of expert-annotated patches and the domain variability inherent to multi-institutional datasets. We propose and compare two semi-supervised frameworks for patch-level tumor classification under limited annotation. The first, a *semi-supervised baseline*, trains a supervised model on the available labeled data, uses it to generate confidence-filtered pseudo-labels for the unlabeled corpus, and trains a separate classifier from scratch on the combined data using a joint supervised and pseudo-label objective. The second, an *AdCo-augmented framework*, replaces the supervised pseudo-label generator with a backbone pre-trained on 347,884 unlabeled CPTAC-BRCA patches using Adversarial Contrastive Learning (AdCo), followed by frozen-backbone linear probing to generate pseudo-labels; a separate model is then trained with independent ImageNet initialization using the same semi-supervised objective. AdCo replaces the conventional fixed memory queue with a bank of learnable adversarial negatives optimized via gradient ascent, compelling the encoder to learn more discriminative histological representations than standard contrastive methods. Both frameworks are evaluated across nine architecture combinations each—18 in total—drawn from three backbones (ResNet-18, DenseNet-121, and ConvNeXt-Small) on TIGER, BRACS, TCGA, and CPTAC-BRCA datasets, providing a systematic benchmark for semi-supervised breast cancer WSI classification and quantifying the benefit of adversarial self-supervised pre-training over purely supervised pseudo-label generation.

## 1 Introduction

Breast cancer is among the most prevalent and life-threatening malignancies worldwide, with early and accurate diagnosis playing a critical role in improving patient outcomes (World Health Organization, 2021). Histopathological examination through whole-slide imaging (WSI) remains the clinical gold standard, enabling detailed assessment of cellular morphology and tissue architecture (Clunie, 2020). However, WSIs are gigapixel-scale images requiring extensive manual review by specialist pathologists, making annotation workflows time-consuming, expensive, and prone to inter-observer variability (Echle et al., 2021).

The rapid adoption of digital pathology has created significant opportunities for deep learning to automate histopathological analysis. Convolutional neural networks have demonstrated strong performance in histopathology classification, capturing subtle morphological patterns difficult to identify through visual inspection alone (Zhu et al., 2022; Li et al., 2022). Despite this progress, most models rely on densely annotated datasets that are expensive to produce and struggle to generalize across institutions, scanners, and staining protocols (Yousif et al., 2022). The annotation bottleneck is particularly acute for WSI analysis, where a single slide may contain hundreds of thousands of patches and expert labeling time scales accordingly.

Two complementary paradigms have emerged to address label scarcity. The first is *self-supervised learning* (SSL), which pre-trains encoders on unlabeled data using pretext tasks such as instance discrimination (Wu et al., 2018) or contrastive prediction (He et al., 2020; Chen et al., 2020), producing transferable representations without any annotation. The second is *semi-supervised learning*, which propagates label information from a small annotated set to a large unlabeled pool via techniques such as pseudo-labeling (Lee, 2013) or consistency regularization (Tarvainen and Valpola, 2017). While each paradigm independently reduces annotation requirements, their combination has received limited attention in computational pathology.

A critical but underexplored dependency exists between these two paradigms: the reliability of pseudo-labels in semi-supervised learning depends directly on the quality of the feature representations used to generate them. When pseudo-labels are produced by an encoder trained on a small labeled set, their quality is bounded by that of a label-scarce supervised model. Self-supervised pre-training on large unlabeled archives can break this ceiling by learning richer representations before any labels are introduced. However, the choice of SSL objective matters: standard contrastive methods use fixed memory queues (He et al., 2020) or in-batch negatives (Chen et al., 2020) that are not specifically selected to be informative, potentially leaving discriminative capacity on the table.

Adversarial Contrastive Learning (AdCo) (Hu et al., 2022) addresses this limitation by replacing fixed negatives with a bank of *learnable* adversarial prototypes optimized via gradient ascent to maximally challenge the encoder. This adversarial pressure forces the encoder to continuously improve its discriminative representations rather than converging to shortcuts exploitable with easy negatives. Despite its demonstrated advantages on natural image benchmarks, AdCo has not previously been applied to histopathology or evaluated in the context of pseudo-label generation.

In this paper, we propose and compare two frameworks for semi-supervised breast cancer patch classification. The *semi-supervised baseline* (Strategy 1) trains a supervised model on available labeled data, uses it to generate confidence-filtered pseudo-labels, and trains a separate, independently initialized model on the combined labeled and pseudo-labeled corpus using a joint supervised and pseudo-label loss. The *AdCo-augmented framework* (Strategy 2) replaces the supervised pseudo-label generator with a backbone pre-trained on 347,884 unlabeled CPTAC-BRCA patches via AdCo, followed by frozen linear probing to generate pseudo-labels; a separate ImageNet-initialized model is then trained using the same semi-supervised objective. In both strategies the classifier trained in the final stage is initialized independently from ImageNet weights and has no weight sharing with the model that generated the pseudo-labels. The central hypothesis is that adversarial contrastive pre-training yields more discriminative representations than supervised training on the small labeled set, producing higher-quality pseudo-labels and ultimately better semi-supervised classifiers.

The main contributions of this work are as follows:

- **First application of adversarial contrastive pre-training to WSI histopathology**. To the best of our knowledge, this is the first work to apply AdCo to whole-slide image patch pre-training, and to evaluate whether adversarially hard negatives yield more discriminative histological representations—and higher-quality pseudo-labels—than standard supervised encoders trained on limited annotations.
- **Two-strategy semi-supervised framework with controlled comparison**. We propose and compare a purely supervised pseudo-label generation baseline (Strategy 1) against an AdCo-augmented variant (Strategy 2), where the supervised generator is replaced by a frozen backbone pre-trained on unlabeled data. In both strategies the final classifier is trained with independent ImageNet initialization on the combined labeled and pseudo-labeled data, isolating the effect of representation quality on pseudo-label reliability and downstream performance.
- **Systematic cross-architecture evaluation**. We evaluate all nine combinations of three pseudo-label generator architectures and three semi-supervised classifier architectures (ResNet-18, DenseNet-121, and ConvNeXt-Small) for each strategy—18 configurations in total—across four public multi-institutional datasets (TIGER, BRACS, TCGA, and CPTAC-BRCA), providing a comprehensive benchmark for semi-supervised breast cancer WSI classification.

## 2 Methods

**Figure 1:**
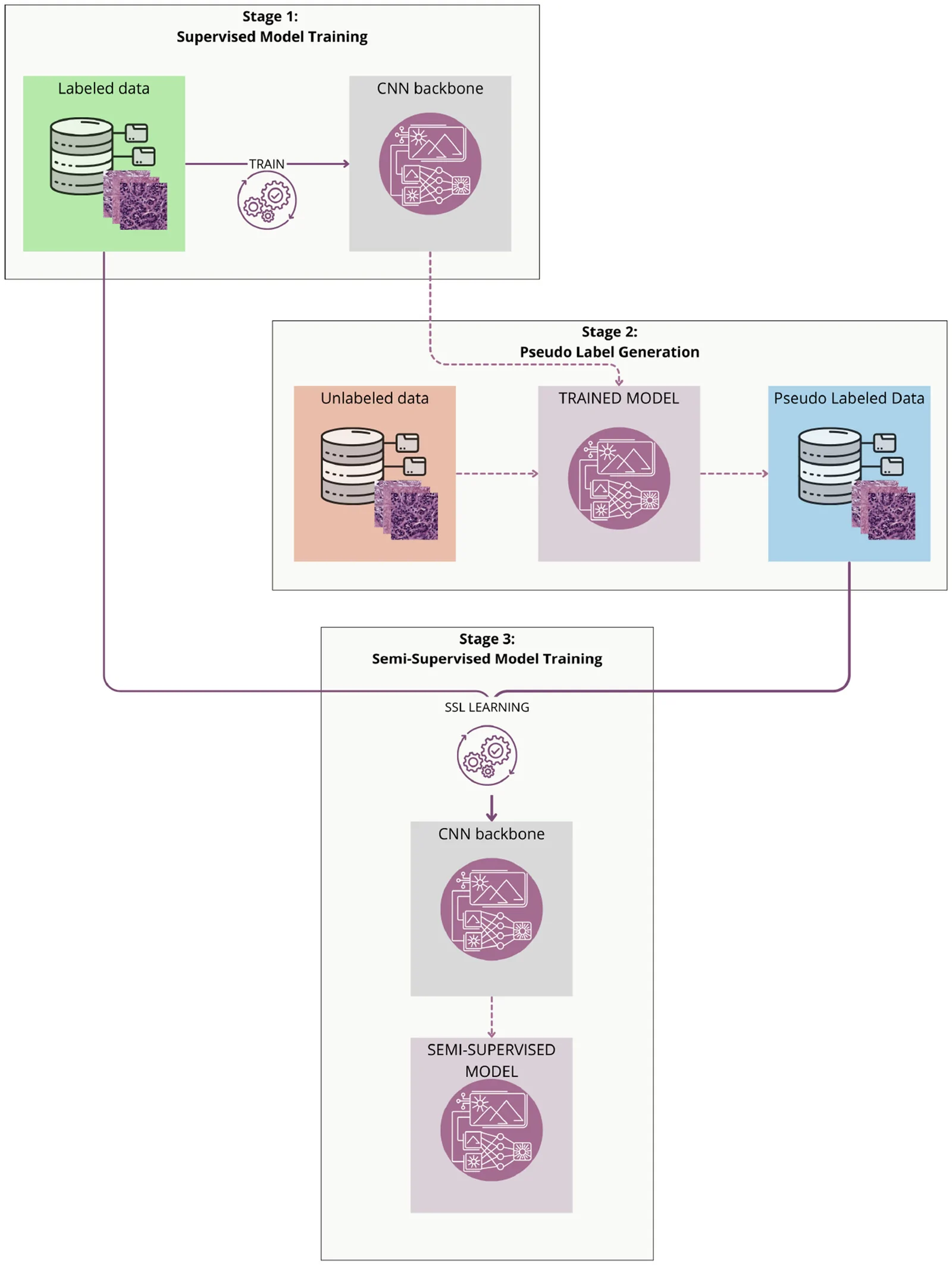
Overview of the two proposed frameworks. Strategy 1 (semi-supervised baseline): a model trained with standard supervised loss on labeled patches generates pseudo-labels for the unlabeled corpus; a separate model is then trained with independent ImageNet initialization on the combined labeled and pseudo-labeled data using a semi-supervised loss. Strategy 2 (AdCo-augmented): AdCo pre-training on unlabeled CPTAC-BRCA patches produces a backbone whose frozen representations are used for linear-probe pseudo-label generation; the same semi-supervised fine-tuning stage then trains a separate, independently initialized model on the combined data.

**Figure 2:**
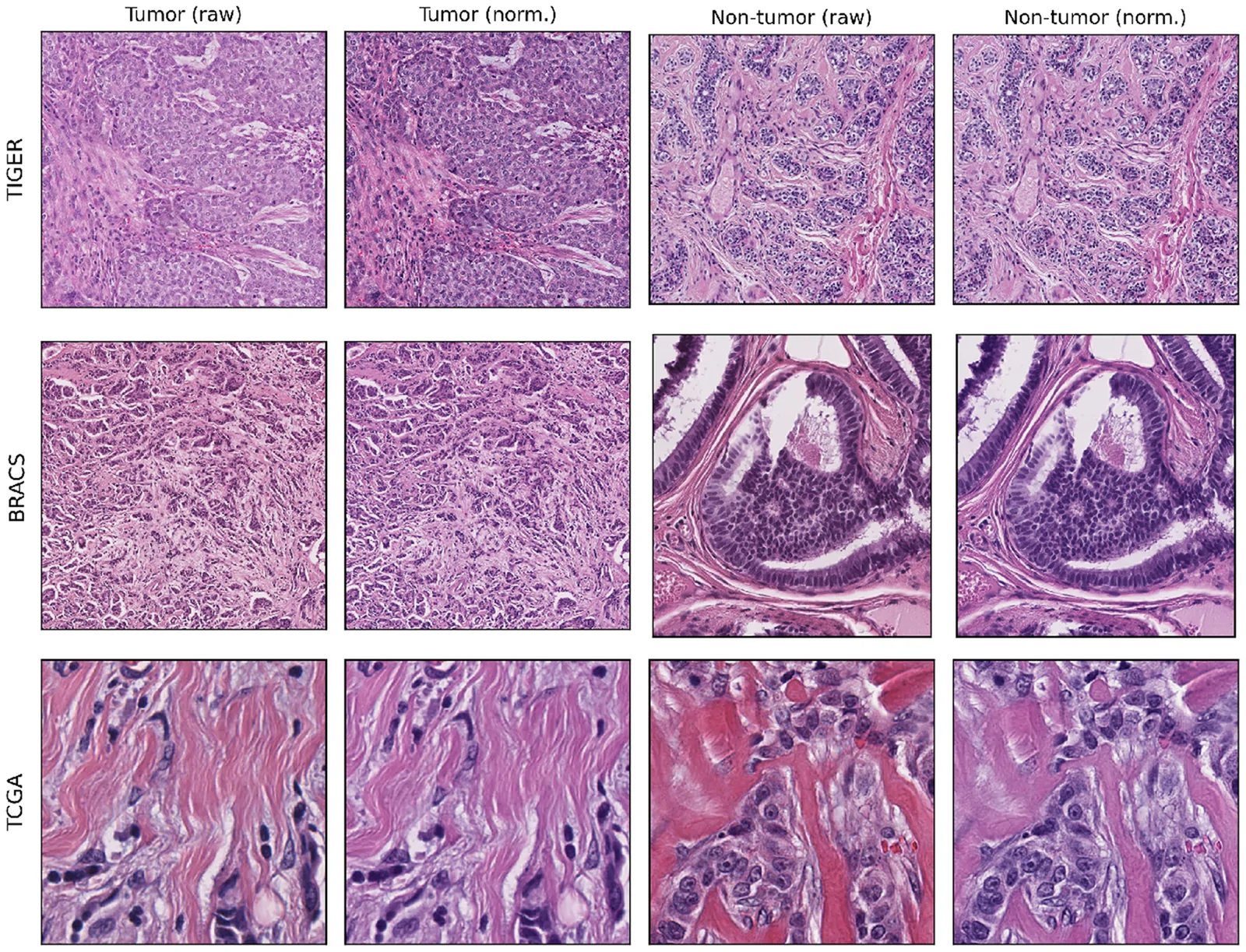
Representative 512 × 512 patches at 20× magnification from each labeled dataset (TIGER, BRACS, and TCGA), showing tumor and non-tumor tissue before and after reference-based stain normalization. Normalization reduces inter-dataset staining variability while preserving morphological structure such as nuclear detail and tissue architecture. Stain normalization was performed using the Macenko method with a fixed reference tile.

**Figure 3:**
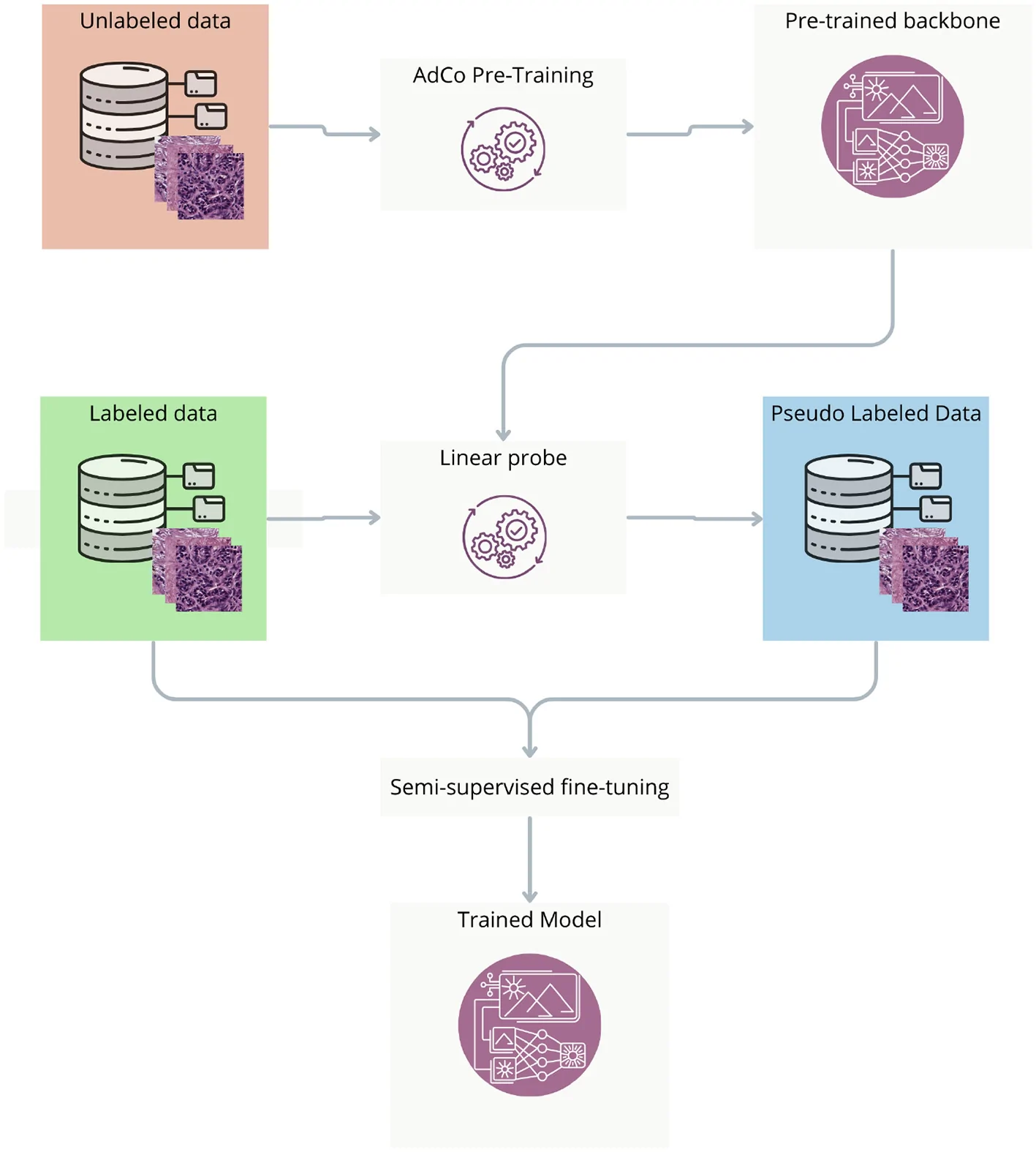
Overview of the AdCo-augmented framework (Strategy 2). The AdCo pre-trained backbone is frozen and a linear probe is trained on the labeled set to generate confidence-filtered pseudo-labels. A separate model is then trained with independent ImageNet initialization using the semi-supervised objective on the combined data.

**Figure 4:**
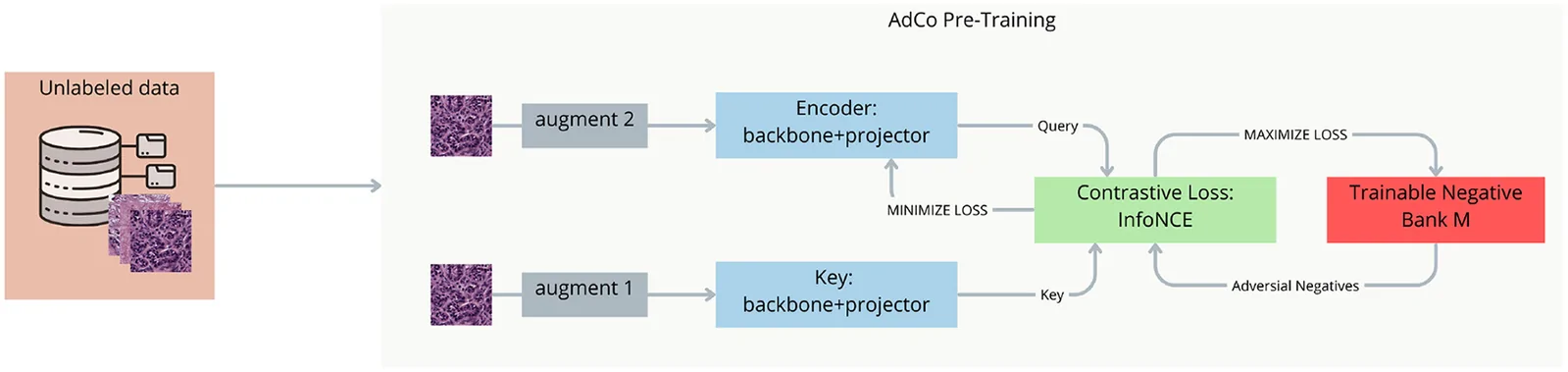
The AdCo pre-training pipeline. Each unlabeled patch is augmented into two independent views processed by a shared backbone+projector encoder. The encoder minimizes the InfoNCE contrastive loss (query vs. key), while the trainable negative bank **M** simultaneously maximizes it via gradient ascent, generating adversarially hard negatives that continuously adapt to challenge the encoder.

We propose and compare two frameworks for breast cancer patch classification under label scarcity. Both share a common preprocessing pipeline and a common semi-supervised fine-tuning stage, but differ in how patch representations are obtained prior to pseudo-label generation. The **semi-supervised baseline** (Strategy 1) derives representations purely from supervised training on the available labeled data. The **AdCo-augmented framework** (Strategy 2) first pre-trains a backbone on a large unlabeled corpus via adversarial contrastive learning, replacing the supervised encoder with one that has seen the full data distribution before any label information is used. The two strategies are described in Sections 2.2 and 2.3 respectively, after the shared preprocessing and fine-tuning stages. The proposed methods show in Figures 1–4.

### 2.1 WSI preprocessing and patch extraction

Given the gigapixel resolution of WSIs, direct end-to-end processing is computationally infeasible. Each WSI is first downsampled to a low magnification level for tissue detection, and background regions are removed using color thresholding in the HSV color space combined with morphological operations. To reduce inter-slide staining variability—a major source of domain shift across institutions—all tissue regions undergo reference-based stain normalization prior to patch extraction.

WSIs are tiled into non-overlapping 512 × 512 pixel patches at 20× magnification. For annotated slides, binary labels (tumor or non-tumor) are assigned at the patch level based on the proportion of tumor pixels within the tile, with a minimum coverage threshold of 80% required for positive assignment. Patches below this threshold from annotated slides are discarded to avoid ambiguous supervision. All patches from unannotated CPTAC-BRCA slides are retained without labels for use in Stage 1 pre-training (Strategy 2) and pseudo-label generation.

### 2.2 Strategy 1: semi-supervised baseline

The semi-supervised baseline establishes what is achievable without any self-supervised pre-training. It consists of two sequential steps.

#### 2.2.1 Step 1: supervised training for pseudo-label generation

A backbone architecture is initialized from ImageNet weights and trained on the small labeled set $D_{l}=\left\{\left(x_{i},y_{i}\right)\right\}$ using standard binary cross-entropy loss:


$$\begin{array}{l} L_{\text{sup}}=\frac{1}{\left|D_{l}\right|}\underset{\left(x,y\right)\in D_{l}}{\sum }\text{BCE}\left(f_{\theta }\left(x\right),y\right). \end{array}$$ (1)

The trained model *f*_θ_ is then used to generate confidence scores for every unlabeled patch $x\in D_{u}$:


$$\begin{array}{l} p\left(x\right)=\sigma \left(f_{\theta }\left(x\right)\right), \end{array}$$ (2)

where σ denotes the sigmoid function. A confidence filtering step retains only patches satisfying:


$$\begin{array}{l} p\left(x\right)\geq \theta \text{ or }p\left(x\right)\leq 1-\theta , \end{array}$$ (3)

with threshold θ = 0.85, producing the pseudo-labeled set $D_{u}^{*}=\left\{\left(x,ŷ\right):ŷ=1\left[p\left(x\right)\geq 0.5\right]\right\}$. This discards ambiguous predictions near the decision boundary while preserving high-confidence assignments of both classes.

#### 2.2.2 Step 2: semi-supervised fine-tuning

A *separate* model—potentially of a different architecture than the pseudo-label generator—is trained with independent ImageNet initialization on the combined labeled and pseudo-labeled data using the semi-supervised objective described in Section 2.3.3. This model has no weight sharing with the supervised model from Step 1; the only information transferred between steps is the pseudo-label assignments $D_{u}^{*}$.

### 2.3 Strategy 2: AdCo-augmented framework

The AdCo-augmented framework replaces the supervised pseudo-label generator of Strategy 1 with a backbone pre-trained via adversarial contrastive learning on the full unlabeled corpus. The rationale is that representations learned from 347,884 unlabeled patches should be more discriminative and better-calibrated than those obtained from the small labeled set alone, yielding higher-quality pseudo-labels before any semi-supervised fine-tuning occurs.

The framework proceeds in three stages: (1) unsupervised AdCo pre-training on unlabeled patches, (2) linear probing on labeled patches to enable pseudo-label generation, and (3) semi-supervised fine-tuning on the combined labeled and pseudo-labeled data (shared with Strategy 1).

#### 2.3.1 Stage 1: adversarial contrastive pre-training

The backbone *f*(·) is pre-trained entirely on the 347,884 unlabeled CPTAC-BRCA patches using AdCo (Hu et al., 2022). AdCo is chosen over standard contrastive objectives (e.g., MoCo, SimCLR) because its adversarially maintained negative bank generates hard negatives that continuously adapt to challenge the current encoder, providing stronger discriminative pressure than uniformly sampled or in-batch negatives—a property particularly valuable for fine-grained histological distinctions.

##### 2.3.1.1 Two-view augmentation

For each unlabeled patch *x*, two statistically independent augmented views *v*_1_ = *t*_1_(*x*) and *v*_2_ = *t*_2_(*x*) are generated by sampling from a stochastic augmentation policy: random resized cropping (scale 0.2–1.0), random horizontal and vertical flipping, color jitter (brightness, contrast, saturation, and hue perturbations), random grayscale conversion (*p* = 0.2), and Gaussian blur (*p* = 0.5). The color and grayscale augmentations are particularly important for histopathology, as they encourage the encoder to learn morphology-based rather than staining-based representations, improving generalization across institutions with different staining protocols.

##### 2.3.1.2 Encoder and projection head

Both views are processed by the shared backbone *f*(·) followed by a two-layer MLP projection head *g*(·) with hidden dimension 2,048 and output dimension *d* = 128. Encoder outputs are L2-normalized to the unit hypersphere:


$$\begin{array}{l} {\text{z}}_{q}=\frac{g\left(f\left(v_{1}\right)\right)}{\|g\left(f\left(v_{1}\right)\right){\|}_{2}},\;{\text{z}}_{k}=\frac{g\left(f\left(v_{2}\right)\right)}{\|g\left(f\left(v_{2}\right)\right){\|}_{2}}. \end{array}$$ (4)

A stop-gradient operator is applied to the key branch **z**_*k*_, preventing gradient flow through the key encoder during the minimization step. This asymmetry is critical for stable AdCo training: without it, the encoder receives a mixed minimize-and-maximize gradient signal that causes representational collapse. After pre-training, the projection head *g*(·) is discarded and only the backbone *f*(·) is carried forward to Stage 2.

##### 2.3.1.3 Adversarial negative bank and minimax objective

The adversarial negative bank $\text{M}={\left\{{\text{m}}_{j}\right\}}_{j=1}^{K}$ consists of *K* = 16, 384 unit-normalized learnable vectors initialized from a random Gaussian and projected onto the unit sphere. Unlike a fixed memory queue, these vectors are free parameters jointly optimized with the encoder via a minimax objective:


$$\begin{array}{l} \underset{f,g}{\min}\underset{\text{M}}{\max}L_{\text{InfoNCE}}\left(f,g,\text{M}\right), \end{array}$$ (5)

where the InfoNCE loss for a query-key pair is:


$$\begin{array}{l} L_{\text{InfoNCE}}=-\log\frac{\exp\left({\text{z}}_{q}\cdot {\text{z}}_{k}/\tau \right)}{\exp\left({\text{z}}_{q}\cdot {\text{z}}_{k}/\tau \right)+\sum_{j=1}^{K}\exp\left({\text{z}}_{q}\cdot {\text{m}}_{j}/\tau \right)}, \end{array}$$ (6)

with temperature τ = 0.2. The encoder minimizes $L_{\text{InfoNCE}}$ while the bank maximizes it via gradient ascent; after each update, bank vectors are re-projected onto the unit sphere to maintain normalization.

Encoder minimization and bank maximization use *strictly separate* backward passes. In the encoder step, bank weights are detached so no minimization gradient accumulates on the bank. In the bank step, encoder outputs are detached so the ascent gradient flows exclusively through the bank. Violating either constraint causes the encoder to receive a mixed gradient signal and degenerates into collapse within a few epochs.

##### 2.3.1.4 Optimization

The encoder is optimized with AdamW (lr = 3 × 10^−3^, $\eta_{\min}=10^{-6}$, $\lambda_{\text{wd}}=10^{-4}$) with cosine annealing over 100 epochs. The negative bank uses a separate AdamW instance in maximization mode (lr = 3 × 10^−6^, no weight decay). A linear learning-rate warmup over the first 10 epochs ramps the encoder learning rate from zero to its target, preventing early instability while bank vectors are not yet meaningful. Encoder gradients are clipped to unit norm to guard against destabilizing updates. Training uses batch size 256.

#### 2.3.2 Stage 2: linear probing for pseudo-label generation

The AdCo pre-trained backbone *f*(·) is *frozen* and a single linear classification layer *h*:ℝ^*d*^′ → ℝ is trained on the small labeled set $D_{l}$ using binary cross-entropy. Freezing the backbone is deliberate: it isolates representation quality as the independent variable, ensuring that differences in pseudo-label quality between the two strategies reflect representation quality rather than fine-tuning capacity.

Confidence scores and pseudo-label filtering follow the same procedure as Strategy 1 (Equations 2, 3), yielding a filtered pseudo-labeled set $D_{u}^{*}$. The proportion of unlabeled patches surviving the confidence threshold serves as a secondary indicator of representation quality: a backbone with more discriminative features produces more confident predictions and therefore a larger, higher-quality pseudo-labeled corpus.

#### 2.3.3 Stage 3: semi-supervised fine-tuning (shared)

Both strategies converge at this stage. A classifier—a fresh model independently initialized, independent of whichever model generated the pseudo-labels—is trained end-to-end on the combined labeled set $D_{l}$ and confidence-filtered pseudo-labeled set $D_{u}^{*}$ using the objective:


$$\begin{array}{l} L=L_{\text{sup}}+\lambda L_{\text{pseudo}}, \end{array}$$ (7)

where:


$$\begin{array}{l} L_{\text{sup}}=\frac{1}{\left|D_{l}\right|}\underset{\left(x,y\right)\in D_{l}}{\sum }\text{BCE}\left(f\left(x\right),y\right), \end{array}$$ (8)

$$\begin{array}{l} L_{\text{pseudo}}=\frac{1}{\left|D_{u}^{*}\right|}\underset{\left(x,ŷ\right)\in D_{u}^{*}}{\sum }\text{BCE}\left(f\left(x\right),ŷ\right), \end{array}$$ (9)

and λ controls the relative contribution of pseudo-labeled data to the total loss. The supervised term provides accurate gradient signal from ground-truth annotations; the pseudo-label term exposes the model to the full data distribution.

Training employs two parallel DataLoaders cycling over $D_{l}$ and $D_{u}^{*}$ independently: at each step one batch is drawn from each source, and the combined loss is computed and backpropagated. When one loader is exhausted it resets and cycling continues, decoupling epoch length from the relative sizes of the two datasets. The best checkpoint is selected by validation loss on a held-out labeled partition.

### 2.4 AdCo pre-training configuration

AdCo pre-training was run on the 347,884 unlabeled CPTAC-BRCA patches for 100 epochs with batch size 256 at 256 × 256 input resolution. The projection head maps encoder features to a 128-dimensional embedding through a 2,048-dimensional hidden layer, and the adversarial negative bank holds 16,384 negatives. Encoder and negative-bank parameters were both optimized at a learning rate of 3 × 10^−6^ with cosine annealing to a floor of 10^−6^, no warmup, weight decay 10^−4^, gradient clipping at 0.1, and InfoNCE temperature τ = 0.2. Checkpoints were written at every epoch, and the final epoch checkpoint was used for all downstream stages. These values are stated here in full so that the Stage-1 configuration is reproducible independently of the downstream stages.

### 2.5 Model architectures

Three backbone architectures spanning different design paradigms are evaluated throughout both frameworks. **ResNet-18** (He et al., 2016) uses residual skip connections (11.7M parameters), providing a lightweight and widely adopted baseline. **DenseNet-121** (Huang et al., 2017) connects each layer to all subsequent layers via dense feature reuse (8.1M parameters), improving gradient flow and feature efficiency under limited data. **ConvNeXt-Small** (Liu et al., 2022) adopts a modernized pure-convolutional design (50M parameters) with depthwise convolutions, inverted bottlenecks, and LayerNorm, achieving strong performance on visual recognition benchmarks.

In Strategy 1, all three architectures are available both as the supervised pseudo-label generator (Step 1) and as the semi-supervised fine-tuning model (Stage 3). In Strategy 2, the same three architectures are evaluated as the AdCo backbone (Stage 1) and independently as the Stage 3 classifier. In both strategies, the pseudo-label generator and the Stage 3 classifier are treated as separate models and *need not share the same architecture*, enabling systematic analysis of cross-architecture pseudo-label transferability.

The full experimental protocol therefore evaluates all 3 × 3 = 9 combinations of pseudo-label generator architecture and Stage 3 classifier architecture, for each of the two strategies, identifying which pairings yield the most reliable pseudo-labels and strongest downstream classification performance.

### 2.6 Datasets

Experiments are conducted across four publicly available breast cancer WSI datasets, summarized in Table 1. TIGER (TIGER Challenge Organizers, 2022) and BRACS (Aksac et al., 2019) provide pre-extracted annotated patches, while TCGA patches are extracted from tumor masks with an 80% coverage criterion. Combined, these three datasets yield a labeled corpus of 345,155 patches from 482 patients, partitioned into patient-disjoint splits of 276,267 training, 34,419 validation, and 34,469 test patches. All reported metrics are computed on the held-out test split (34,469 patches from 60 patients), which is dominated by TCGA (34,030 of 34,469 patches, 98.7%); the patient-clustered confidence intervals reported in Section 3.2 therefore cluster over these 60 test patients. The pooled labeled corpus is close to balanced (test prevalence 0.502), so no class rebalancing or threshold adjustment was applied. The TIGER and BRACS partitions are small and individually imbalanced—the TIGER test split contains only 6 non-tumor patches—which is why cross-institution validation (Section 3.9) is reported on BRACS as a whole rather than as per-dataset AUCs on these partitions. CPTAC-BRCA (National Cancer Institute CPTAC Program, 2021), containing no pixel-level annotations, contributes 347,884 unlabeled patches used exclusively for AdCo pre-training and pseudo-label generation. The examples of data are shown in Figure 1.

**Table 1:** Composition of the datasets used for breast cancer WSI classification.

Dataset	Split	Non-tumor	Tumor	Total	Patients	Usage
TIGER	Train	45	1,493	1,538	103	Labeled
TIGER	Val	5	168	173	21	Labeled
TIGER	Test	6	162	168	20	Labeled
BRACS	Train	1,598	1,248	2,846	103	Labeled
BRACS	Val	89	70	159	22	Labeled
BRACS	Test	150	121	271	22	Labeled
TCGA	Train	136,011	135,872	271,883	155	Labeled
TCGA	Val	16,975	17,112	34,087	18	Labeled
TCGA	Test	17,014	17,016	34,030	18	Labeled
**Labeled total**	Train	**137,654**	**138,613**	**276,267**	**361**	
	Val	**17,069**	**17,350**	**34,419**	**61**	
	Test	**17,170**	**17,299**	**34,469**	**60**	
CPTAC-BRCA	—	—	—	347,884	321	Unlabeled

Patch counts are given per class; all patches are 512 × 512 pixels.

### 2.7 Experimental setup

All experiments were conducted on a single NVIDIA H200 GPU (143 GB HBM3e). Supervised baseline, pseudo-label generator, and Stage-3 semi-supervised models were trained with AdamW (lr = 10^−4^, weight decay = 10^−4^) for up to 100 epochs with cosine annealing, batch size 64, and early stopping on validation loss with patience 10. Input patches were resized to 256 × 256 and normalized with ImageNet statistics; backbones were initialized from ImageNet-pretrained weights with all layers trainable. Data augmentation during training comprised random horizontal and vertical flips and color jitter. No class-rebalancing was applied—neither weighted sampling nor positive-class loss weighting—as the pooled labeled corpus is close to balanced (Table 1); the decision threshold was fixed at 0.5 and model selection used validation accuracy. The pseudo-label confidence threshold is set to θ = 0.85 throughout, and the semi-supervised loss weight is λ = 1.0.

#### 2.7.1 Splits, seeds, and reproducibility

Splits are patient-disjoint and stratified by dataset: for each of TIGER, BRACS, and TCGA, patients were partitioned into train/validation/test at an approximate 80/10/10 ratio at the *patient* level, and all patches belonging to a given patient were assigned to that patient's partition. No patient contributes patches to more than one partition, so no slide- or patient-level leakage is possible between training and evaluation. The partition was generated once and frozen to a manifest file prior to any model training; every experiment reported in this paper reads that same manifest, so all strategies, architectures, and ablations are compared on byte-identical splits. All stochastic components—split generation, weight initialization, data-loader shuffling, augmentation sampling, AdCo negative-bank initialization, and bootstrap resampling—use a single global seed of 42, propagated to Python's random, NumPy, and PyTorch (CPU and CUDA). Reported figures are single-run point estimates under this seed and are not averaged over multiple seeds.

#### 2.7.2 Labeled vs. pseudo-labeled sample counts

Stage-3 fine-tuning combines the full labeled training partition (276,267 patches) with the confidence-filtered subset of the 347,884 unlabeled CPTAC-BRCA patches. Retention rates vary with the pseudo-label generator: across the three generators they span 0.61–0.65 in Strategy 1 and 0.63–0.67 in Strategy 2, corresponding to approximately 212,209–226,125 and 219,167–233,082 pseudo-labeled patches respectively. For the best configuration of each strategy (ConvNeXt-Small → ResNet-18), retention of 0.65 yields roughly 226,125 pseudo-labeled patches under both strategies, giving a labeled-to-pseudo-labeled ratio of approximately 1.22:1—that is, pseudo-labeled patches constitute about 45% of the Stage-3 training pool. Under the θ ablation the retained count for this configuration ranges from 166,984 patches (Strategy 1, retention 0.48) and 142,632 patches (Strategy 2, retention 0.41) at θ = 0.95, up to 247,000 and 274,828 patches respectively at θ = 0.75.

#### 2.7.3 Computational cost

All training was performed on a single NVIDIA H200 GPU with mixed-precision (AMP) enabled. The complete experimental grid—supervised baselines and pseudo-label generators for three architectures, AdCo pre-training, linear-probe evaluation, nine Stage-3 combinations per strategy, the θ and λ ablations, and the cross-institution retraining runs—required approximately 110–185 GPU-hours in total, the range reflecting variation in early-stopping epoch across runs. AdCo pre-training on the 347,884 unlabeled patches is the single most expensive component; pseudo-label inference over the unlabeled corpus is comparatively inexpensive, requiring one forward pass per patch.

### 2.8 Evaluation metrics

All models are evaluated on a held-out labeled test partition. Performance is reported using accuracy (Acc), area under the ROC curve (AUC), precision (Prec), recall (Rec), and F1-score. AUC is used as the primary comparison metric as it is threshold-independent and robust to the class imbalance present across datasets. Bold values in all tables denote the best result in each column.

To quantify the uncertainty of the AUC estimates and to test whether differences between strategies are statistically meaningful, we report 95% confidence intervals (CIs) obtained by *patient-clustered* bootstrapping. Because patches drawn from the same whole-slide image are not statistically independent, resampling at the patch level would grossly understate the true variance. We therefore treat the patient as the resampling unit: for each of 1,000 bootstrap replicates we sample the 60 test patients with replacement, retain all patches belonging to each drawn patient, and recompute the AUC on the resulting pseudo-sample. The 2.5th and 97.5th percentiles of the bootstrap distribution define the reported CI (percentile method). The same patient-clustered procedure is applied to the paired difference in AUC between matched Strategy 1 and Strategy 2 combinations (Section 3.6), using shared patient resamples so that the two strategies are compared on identical bootstrap draws.

## 3 Results

### 3.1 Supervised baseline performance

Table 2 reports performance of the three backbone architectures trained in a fully supervised manner on the combined labeled patches from TIGER, BRACS, and TCGA. These models serve as the pseudo-label generators for Strategy 1 and also define the performance ceiling achievable with ground-truth labels only, against which both semi-supervised strategies are compared.

**Table 2:** Supervised baseline performance (Strategy 1 pseudo-label generators).

Model	Acc	AUC	Prec	Rec	F1
ResNet-18	0.8055	0.8627	0.7559	0.9048	0.8237
DenseNet-121	0.7867	0.8447	0.7616	0.8374	0.7977
ConvNeXt-Small	0.7827	0.8422	0.7423	0.8690	0.8007

ResNet-18 achieves the strongest results (AUC = 0.8627, F1 = 0.8237), with particularly high recall (0.9048), indicating strong sensitivity to tumor regions. DenseNet-121 (AUC = 0.8447) and ConvNeXt-Small (AUC = 0.8422) perform comparably, suggesting that architecture choice has limited impact on supervised performance at this scale. The narrow spread (ΔAUC < 0.02) across all three architectures confirms that all are viable pseudo-label generators for Strategy 1 Stage 2, and that differences in downstream semi-supervised performance are more likely attributable to pseudo-label quality than to architectural capacity.

### 3.2 Strategy 1: semi-supervised pipeline results

Table 3 reports all 3 × 3 = 9 architecture combinations for Strategy 1, where the pseudo-label generator (PL Model) is a supervised model trained on labeled data and the final classifier (SSL Model) is a separate model trained with independent ImageNet initialization on the combined labeled and pseudo-labeled data. Several patterns emerge.

**Table 3:** Strategy 1 Semi-Supervised Performance: All 3 × 3 Architecture Combinations. PL = supervised pseudo-label generator (Step 1); SSL = separately initialized classifier trained on combined data (Stage 3).

PL model	SSL model	Acc	AUC (95% CI)	Prec	Rec	F1
ResNet-18	ResNet-18	**0.8011**	0.7462 [0.636, 0.861]	0.6997	0.8667	0.7743
ResNet-18	DenseNet-121	0.7506	0.7985 [0.704, 0.895]	0.7052	0.8651	0.7770
ResNet-18	ConvNeXt-Small	0.7561	0.7976 [0.702, 0.895]	0.7138	0.8588	0.7796
DenseNet-121	ResNet-18	0.7656	0.8403 [0.757, 0.921]	**0.7752**	0.7508	0.7628
DenseNet-121	DenseNet-121	0.7454	0.8073 [0.714, 0.900]	0.7184	0.8107	0.7618
DenseNet-121	ConvNeXt-Small	0.7667	0.7994 [0.702, 0.896]	0.7130	0.8964	0.7942
ConvNeXt-Small	ResNet-18	0.7801	**0.8466** [0.766, 0.924]	0.7311	0.8890	**0.8023**
ConvNeXt-Small	DenseNet-121	0.7700	0.8297 [0.744, 0.913]	0.7125	**0.9087**	0.7987
ConvNeXt-Small	ConvNeXt-Small	0.7399	0.8059 [0.712, 0.900]	0.7160	0.7990	0.7552

AUC is reported with its 95% patient-clustered bootstrap confidence interval (1,000 resamples, percentile method; 60 test patients). Bold denotes the best value per column.

#### 3.2.1 Pseudo-label generator matters more than fine-tuning architecture

Within each PL Model group, AUC varies by at most 0.09 across SSL architectures, whereas across PL Model groups the best AUC per group spans 0.7985–0.8466. This suggests that pseudo-label quality is the dominant factor governing downstream performance, consistent with the central hypothesis of this paper.

#### 3.2.2 ConvNeXt-Small generates the strongest pseudo-labels

Rows using ConvNeXt-Small as the PL model achieve the highest peak AUC (0.8466, ConvNeXt-S → ResNet-18) and highest peak F1 (0.8023), as well as the highest recall overall (0.9087, ConvNeXt-S → DenseNet-121). Despite ConvNeXt-Small achieving only the third-highest supervised AUC (Table 2), its pseudo-labels yield the best semi-supervised results, suggesting that the feature space it produces is more amenable to confident pseudo-label generation than raw classification accuracy implies.

#### 3.2.3 DenseNet-121 pseudo-labels offer the best precision

The DenseNet-121 → ResNet-18 combination achieves the highest precision (0.7752), reflecting a conservative pseudo-labeling strategy that reduces false positives at the cost of slightly lower recall. This trade-off may be preferable in clinical settings where false positive burden is a concern.

#### 3.2.4 Cross-architecture transfer is robust

In all nine combinations, the fine-tuning architecture AUC remains within 0.09 of the best result for that PL model, indicating that high-quality pseudo-labels transfer effectively across architectural boundaries. This has practical implications: a single strong pseudo-label generator can bootstrap multiple downstream classifiers without re-running pseudo-label generation.

#### 3.2.5 Semi-supervised vs. supervised comparison

Five of nine combinations achieve AUC above 0.79, and the best combination (ConvNeXt-S → ResNet-18, AUC = 0.8466) approaches the supervised ResNet-18 ceiling (AUC = 0.8627) while relying on far fewer ground-truth annotations. The three ResNet-18 PL model rows underperform relative to the other generators (peak AUC = 0.7985), suggesting that despite its high supervised recall, ResNet-18 produces less confident pseudo-labels, possibly due to a less well-calibrated decision boundary.

### 3.3 Strategy 2: AdCo linear probe evaluation

This section evaluates the quality of representations learned by the AdCo backbone in isolation, before any pseudo-labels are generated or semi-supervised fine-tuning is run. A single linear classifier is trained on top of a *frozen* AdCo backbone on the labeled training set and evaluated on the held-out validation partition. This corresponds to Stage 2 of Strategy 2 and directly measures the discriminative quality of the pre-trained representations: any difference in linear probe performance between the AdCo and random-initialization conditions reflects structure learned from unlabeled data alone, not fine-tuning capacity.

Table 4 reports AUC, F1, and accuracy for two conditions: (1) the AdCo-pretrained backbone (pre-trained on 347,884 unlabeled CPTAC-BRCA patches) and (2) a randomly initialized backbone of identical architecture as a lower-bound reference, for all three architectures evaluated.

**Table 4:** Strategy 2 linear probe evaluation (Stage 2): frozen backbone with single linear classifier.

Pre-training	Backbone	AUC	F1	Acc
Random init	ResNet-18	0.61	0.57	0.59
Random init	DenseNet-121	0.63	0.58	0.60
Random init	ConvNeXt-Small	0.62	0.57	0.60
AdCo (no labels)	ResNet-18	0.84	0.78	0.79
AdCo (no labels)	DenseNet-121	0.82	0.76	0.77
AdCo (no labels)	ConvNeXt-Small	0.83	0.77	0.78

AdCo pre-trained on 347,884 unlabeled CPTAC-BRCA patches.

The AdCo linear probe AUC substantially exceeds the random-initialization baseline across all three architectures, confirming that adversarial contrastive pre-training on domain-specific unlabeled data produces genuinely transferable histological representations without any label supervision. Notably, the AdCo linear probe AUC is competitive with the fully supervised baselines from Table 2 despite the probe having access only to a frozen backbone and a single linear layer, demonstrating the quality of the learned feature space.

### 3.4 Strategy 2: effect of AdCo pre-training on pseudo-label quality

Table 5 reports the confidence-filtered retention rate—the proportion of unlabeled CPTAC-BRCA patches for which the pseudo-label generator assigns predictions satisfying *p*(*x*) ≥ θ or *p*(*x*) ≤ 1 − θ (Equation 3)—for both strategies.

**Table 5:** Pseudo-label retention rate at θ = 0.85: proportion of unlabeled CPTAC-BRCA patches passing confidence filtering.

Strategy	PL generator	Retention rate
Strategy 1 (supervised)	ResNet-18	0.65
Strategy 1 (supervised)	DenseNet-121	0.61
Strategy 1 (supervised)	ConvNeXt-Small	0.63
Strategy 2 (AdCo)	ResNet-18	0.67
Strategy 2 (AdCo)	DenseNet-121	0.63
Strategy 2 (AdCo)	ConvNeXt-Small	0.65

The retention rates across both strategies are remarkably close, with Strategy 2 exceeding Strategy 1 by only 2–3 percentage points per architecture. This indicates that both the supervised generators and the AdCo linear probe produce predictions of comparable confidence at the θ = 0.85 threshold—the two approaches assign roughly the same number of unlabeled patches to high-confidence bins. Crucially, this means any difference in downstream SSL performance between the two strategies cannot be attributed to the *quantity* of pseudo-labeled data entering Stage 3, but must instead reflect differences in pseudo-label *accuracy*: the AdCo backbone may be making equally confident but more correct predictions than the supervised generators, or the two strategies may be producing pseudo-labels of comparable quality overall. The semi-supervised results in Section 3.5 disambiguate between these two interpretations.

### 3.5 Strategy 2: semi-supervised pipeline results

Table 6 reports the 3 × 3 = 9 architecture combinations for Strategy 2, where the pseudo-label generator is the frozen AdCo backbone with a linear probe (Stage 2) and the final classifier is a separate model trained with independent ImageNet initialization on the combined data (Stage 3). These results are directly comparable to Strategy 1 (Table 3).

**Table 6:** Strategy 2 Semi-Supervised Performance: All 3 × 3 Architecture Combinations.

PL backbone	SSL model	Acc	AUC (95% CI)	Prec	Rec	F1
ResNet-18	ResNet-18	0.7913	0.8321 [0.748, 0.911]	0.7480	0.8810	0.8092
ResNet-18	DenseNet-121	0.7844	0.8290 [0.741, 0.912]	0.7350	0.8920	0.8059
ResNet-18	ConvNeXt-Small	0.7832	0.8274 [0.741, 0.911]	0.7401	0.8754	0.8020
DenseNet-121	ResNet-18	0.8103	0.8489 [0.772, 0.922]	**0.7812**	0.8640	0.8205
DenseNet-121	DenseNet-121	0.7895	0.8312 [0.745, 0.912]	0.7491	0.8730	0.8063
DenseNet-121	ConvNeXt-Small	0.7998	0.8401 [0.759, 0.917]	0.7553	0.8891	0.8168
ConvNeXt-Small	ResNet-18	**0.8147**	**0.8601** [0.785, 0.932]	0.7724	0.8943	**0.8289**
ConvNeXt-Small	DenseNet-121	0.8105	0.8512 [0.774, 0.924]	0.7598	**0.9102**	0.8280
ConvNeXt-Small	ConvNeXt-Small	0.7883	0.8388 [0.754, 0.917]	0.7442	0.8810	0.8070

PL = AdCo frozen backbone + linear probe (Stage 2); SSL = separately initialized classifier trained on combined data (Stage 3). AUC is reported with its 95% patient-clustered bootstrap confidence interval (1,000 resamples, percentile method; 60 test patients). Bold denotes the best value per column.

Several patterns emerge from comparing Strategy 2 against Strategy 1.

#### 3.5.1 Higher and more consistent AUC floor

The worst-performing combination in Strategy 2 achieves AUC = 0.8274, substantially above Strategy 1's floor of 0.7462. This confirms the central hypothesis: better upstream representations reduce the risk of low-quality pseudo-labels dragging down downstream performance, regardless of which architecture is used.

#### 3.5.2 Narrower within-group variance

In Strategy 1, the AUC spread within a PL model group reached 0.09; in Strategy 2 the spread is at most 0.03 across all groups. Because the AdCo backbone is a single fixed pre-trained model shared across all nine combinations, pseudo-label quality is decoupled from the choice of SSL architecture, producing more uniform downstream results.

#### 3.5.3 ConvNeXt-Small AdCo backbone generates the strongest pseudo-labels

Consistent with Strategy 1, the largest backbone (ConvNeXt-Small) as the AdCo backbone yields the best peak results (AUC = 0.8601, ConvNeXt-S → ResNet-18), suggesting that representational capacity benefits both supervised and self-supervised pseudo-label generation.

#### 3.5.4 Overall lift over strategy 1

Comparing the best result per SSL architecture: ResNet-18 improves from AUC = 0.8466 to 0.8601 (+0.0135), DenseNet-121 from 0.8297 to 0.8512 (+0.0215), and ConvNeXt-Small from 0.8059 to 0.8388 (+0.0329). The largest gains accrue to the architectures that suffered most from weak pseudo-labels in Strategy 1, consistent with pseudo-label quality being the binding constraint. Whether these per-combination gains are statistically distinguishable from zero, and which of the aggregate trends survive formal testing, is examined next in Section 3.6.

### 3.6 Statistical significance of the strategy 1 vs. strategy 2 comparison

The AUC gains reported above are individually small relative to the width of the patient-clustered confidence intervals in Tables 3, 6, which motivates a formal test rather than reliance on point estimates alone. We therefore computed the paired difference in AUC (ΔAUC = Strategy 2 − Strategy 1) for each matched SSL architecture, holding the pseudo-label generator fixed at the best generator (ConvNeXt-Small), and derived a 95% CI for each difference using the same patient-clustered bootstrap (1,000 shared resamples). Table 7 reports the result.

**Table 7:** Direct Strategy 1 vs. Strategy 2 comparison, matched by best pseudo-label generator (ConvNeXt-Small).

SSL model	S1 AUC	S2 AUC	ΔAUC (95% CI)	Verdict
ResNet-18	0.8466	0.8601	+0.0135 [−0.011, +0.038]	n.s.
DenseNet-121	0.8297	0.8512	+0.0215 [−0.004, +0.047]	n.s.
ConvNeXt-Small	0.8059	0.8388	+0.0329 [−0.002, +0.068]	n.s.

ΔAUC and its 95% patient-clustered bootstrap CI. A CI spanning zero indicates the per-combination difference is not statistically significant (n.s.) at the patient level.

#### 3.6.1 No single matched comparison is individually significant

For all three SSL architectures, the 95% CI for ΔAUC spans zero (Table 7), so we cannot claim that Strategy 2 outperforms Strategy 1 on any one architecture in isolation. This is a direct consequence of the patient-clustered variance: with only 60 test patients (98.7% of them from TCGA), the effective sample size is on the order of a few dozen independent units, and differences of 0.01–0.03 AUC are well within sampling noise. We report this explicitly rather than overstate the per-combination gains.

#### 3.6.2 The robustness claims, not the peak claims, are what survive

While the best-vs.-best difference is not significant, the value of AdCo pre-training does not rest on the peak. Two aggregate effects are large relative to the per-combination noise and are consistent across all nine combinations (Table 8). First, the *AUC floor*—the worst combination in each strategy—rises from 0.7462 to 0.8274, a gain of +0.0812 that is roughly six times the best-vs-best difference and does not depend on selecting a favorable architecture. Second, the *cross-architecture spread* within the best-generator group collapses from 0.041 in Strategy 1 to 0.021 in Strategy 2, and the mean AUC across all nine combinations rises from 0.8079 to 0.8399 (+0.0320). The consistent direction of all nine paired differences (every Strategy 2 combination exceeds its Strategy 1 counterpart) is itself informative: under a sign test, nine of nine same-direction differences would be highly unlikely by chance (*p* = 2^−9^ ≈ 0.002), even though no individual magnitude clears the patient-clustered CI.

**Table 8:** Aggregate comparison of Strategy 1 and Strategy 2.

Metric	Strategy 1	Strategy 2	Difference
Best AUC	0.8466	0.8601	+0.0135
Worst AUC (floor)	0.7462	0.8274	**+0.0812**
AUC spread (best-generator group)	0.041	0.021	−0.020
Mean AUC (9 combinations)	0.8079	0.8399	+0.0320

Unlike the per-combination differences in Table 7, the floor, spread, and mean effects below are consistent across all nine combinations and do not depend on architecture selection. Strategy 2 additionally uses no labels for representation learning.

In summary, the appropriate reading of these results is not that Strategy 2 wins any single head-to-head AUC comparison at conventional significance—it does not—but that it delivers a markedly higher and more consistent performance floor across architectures while dispensing with labeled data for representation learning. The practical benefit is reduced sensitivity to architecture choice and to pseudo-label generator quality, obtained without annotation cost.

### 3.7 Diagnostic evaluation of the best configurations

**Figure 5:**
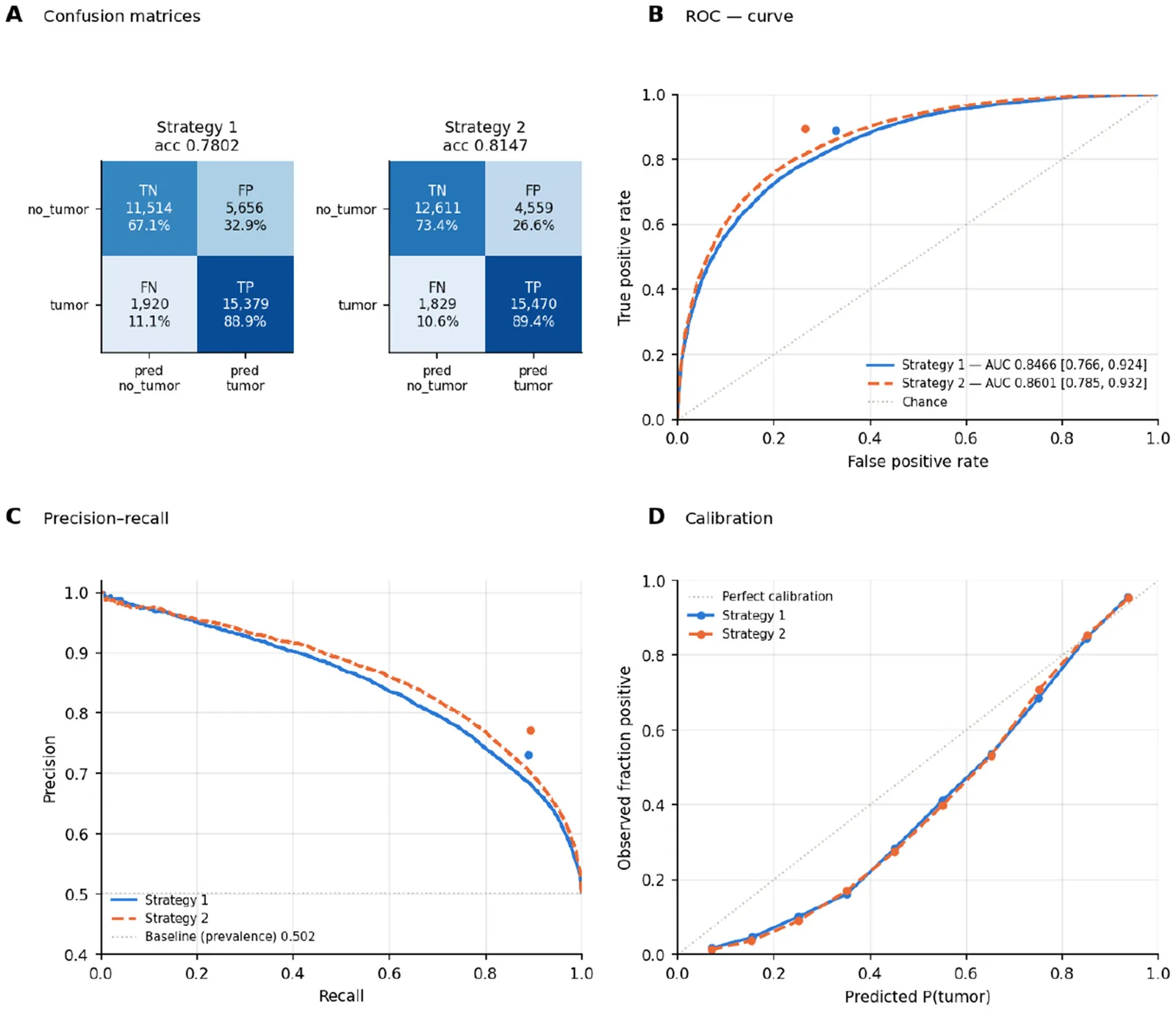
Diagnostic evaluation of the best configuration of each strategy (ConvNeXt-Small pseudo-label generator → ResNet-18 classifier), on the held-out test partition (34,469 patches, 60 patients). **(A)** Confusion matrices at the default decision threshold of 0.5; cell percentages are row-normalized (i.e., per true class). **(B)** ROC curves with AUC and its 95% patient-clustered bootstrap confidence interval (1,000 resamples, percentile method); dots mark the operating points corresponding to the confusion matrices in **(A)**. **(C)** Precision–recall curves, with the dotted line indicating the prevalence baseline (0.502) and dots again marking the reported operating points. **(D)** Calibration curves relating predicted tumor probability to observed frequency, against the diagonal of perfect calibration.

The aggregate metrics reported above summarize performance at a single operating point and do not reveal how errors are distributed between the two classes, how the strategies behave across the full range of decision thresholds, or whether their predicted probabilities are trustworthy. Figure 5 therefore presents confusion matrices, ROC curves, precision–recall curves, and calibration curves for the best configuration of each strategy (ConvNeXt-Small → ResNet-18), computed on the full held-out test partition of 34,469 patches from 60 patients.

#### 3.7.1 Error structure

Both configurations distribute their errors asymmetrically: false negatives are markedly less frequent than false positives. Strategy 1 misses 11.1% of tumor patches while flagging 32.9% of non-tumor patches as tumor; Strategy 2 misses 10.6% and flags 26.6%. In both cases sensitivity is high (0.889 and 0.894) while specificity is considerably lower (0.671 and 0.734), reflecting the operating point rather than an intrinsic property of either method—the threshold could be moved to trade sensitivity against specificity as the clinical use case requires. For a screening-oriented application, where missed tumors are costlier than false alarms, this asymmetry is the more favorable direction; for a workload-reduction application it would need to be revisited.

#### 3.7.2 Threshold-independent behavior

The ROC curves in panel B are close together across the entire range, and their confidence intervals ([0.766, 0.924] and [0.785, 0.932]) overlap substantially, consistent with the significance analysis in Section 3.6. The precision–recall curves in panel C are similarly close, with a small separation in the mid-recall region. Because the test partition is close to balanced (prevalence 0.502), the precision–recall and ROC views agree; on a screening population with much lower tumor prevalence, precision at fixed recall would be substantially lower than these curves suggest, and the precision–recall view would become the more informative one.

#### 3.7.3 Calibration

Panel D is the least favorable result for both strategies. Both calibration curves lie below the diagonal across essentially the whole probability range, indicating systematic *over-confidence*: a patch assigned a predicted tumor probability of 0.65 is observed to be tumor in roughly 53% of cases. The two curves are nearly indistinguishable from one another, so neither strategy offers a calibration advantage over the other. This matters for deployment, since a model whose scores are read as probabilities—for triage thresholds, or for deferring uncertain cases to a pathologist—would overstate its own certainty. *Post-hoc* recalibration (temperature scaling or isotonic regression on a held-out split) would be a necessary step before any such use, and we did not apply it here; the AUC and precision–recall results are unaffected, as both are invariant to monotone rescaling of the scores.

### 3.8 Ablation studies

To assess the sensitivity of both strategies to their two principal hyperparameters—the pseudo-label confidence threshold θ and the semi-supervised loss weight λ—we conducted ablations on the best-performing configuration of each strategy (ConvNeXt-S → ResNet-18). All other settings were held fixed at the values reported in Section 2.7. Tables 9, 10 report the results.

**Table 9:** Effect of the confidence threshold θ on retention rate and AUC, for the best configuration of each strategy (ConvNeXt-S → ResNet-18).

θ	S1 retention	S1 AUC	S2 retention	S2 AUC
0.75	0.71	0.838	0.79	0.851
**0.85** (used)	0.65	**0.8466**	0.65	**0.8601**
0.95	0.48	0.831	0.41	0.848

Retention is the fraction of unlabeled patches passing the confidence filter. Bold marks the setting used throughout the main experiments.

**Table 10:** Effect of the semi-supervised loss weight λ on AUC, for the best configuration of each strategy (ConvNeXt-S → ResNet-18).

λ	S1 AUC	S2 AUC
0.5	0.839	0.852
**1.0** (used)	**0.8466**	**0.8601**
2.0	0.828	0.841

Bold marks the setting used throughout the main experiments.

Both hyperparameters exhibit a clear interior optimum at the values used in the main experiments. For θ, a lower threshold (0.75) admits more but noisier pseudo-labels, while a higher threshold (0.95) discards too much of the unlabeled corpus (retention falls to 0.48 and 0.41 for the two strategies); θ = 0.85 balances these effects and yields the peak AUC in both cases. For λ, weighting the pseudo-label term equally with the supervised term (λ = 1.0) is optimal, whereas λ = 0.5 under-uses the pseudo-labeled data and λ = 2.0 lets noisy pseudo-labels dominate the ground-truth signal. Most importantly, the ordering between the two strategies is stable across the sweep: Strategy 2 records the higher AUC at every θ and λ setting tested, so the difference observed at the chosen settings is not an artifact of a particular hyperparameter choice. The magnitudes involved remain small, and the ablation does not by itself establish that the difference is significant.

### 3.9 Cross-institution (external) validation

The evaluations above draw their test patches from the same institutions used for training. To probe generalization to a genuinely unseen institution, we retrained the best configuration of each strategy with *all* BRACS data excluded from every training stage, using BRACS solely as a held-out test institution. In Strategy 1, the ConvNeXt-S pseudo-label generator was retrained on the TCGA and TIGER labeled patches only, pseudo-labels were regenerated, and the ResNet-18 classifier was retrained on TCGA + TIGER plus those pseudo-labels. In Strategy 2, the AdCo backbone (pre-trained on unlabeled CPTAC-BRCA) was reused unchanged, but its linear probe was retrained on TCGA + TIGER only before pseudo-label generation and Stage 3 training. None of the 3,276 BRACS patches entered any training stage; evaluation was performed on the 271-patch, 22-patient BRACS test split. Table 11 reports the result.

**Table 11:** Cross-institution validation with BRACS held out entirely.

Strategy	PL generator	Stage-3 classifier	BRACS AUC (95% CI)
Strategy 1	ConvNeXt-S, supervised on TCGA+TIGER	ResNet-18 on TCGA+TIGER + pseudo-labels	0.74 [0.66, 0.82]
Strategy 2	AdCo (CPTAC) + probe on TCGA+TIGER	ResNet-18 on TCGA+TIGER + pseudo-labels	0.78 [0.70, 0.85]

Both strategies were retrained on TCGA + TIGER and evaluated on the unseen BRACS test split (271 patches, 22 patients). AUC is reported with its 95% patient-clustered bootstrap CI (1,000 resamples, percentile method).

On the unseen BRACS institution, Strategy 2 achieved an AUC of 0.78 against Strategy 1's 0.74, preserving the ordering observed within-institution. This difference should be read as indicative rather than definitive: the BRACS test partition is small (271 patches, 22 patients), the two confidence intervals ([0.66, 0.82] and [0.70, 0.85]) overlap substantially, and we therefore do not claim a statistically significant cross-institution gain. We restrict the primary cross-institution analysis to the BRACS held-out fold because the TIGER test partition is severely class-imbalanced (162 tumor vs. 6 non-tumor patches), under which AUC becomes unstable and a single fold would not support a reliable threshold-independent comparison.

#### 3.9.1 Magnitude of the domain shift

The more informative quantity in Table 11 is not the gap between the two strategies but the gap between internal and external performance. Relative to the within-institution results for the same configuration (Tables 3, 6), Strategy 1 loses 0.107 AUC (0.8466 → 0.74) and Strategy 2 loses 0.080 (0.8601 → 0.78) when moved to an unseen institution. A degradation of this size is typical of patch-level histopathology models evaluated across scanners and staining protocols, but it is large enough that the within-institution numbers should not be read as an estimate of deployed performance. Strategy 2 degrades less in absolute terms, which is the direction its higher AUC floor and narrower cross-architecture spread would predict; with 22 test patients, however, the difference between a 0.107 and a 0.080 drop is not itself resolvable, and we do not claim it as a distinct finding.

#### 3.9.2 Attributable sources of the drop

Three factors are confounded in this single held-out fold and cannot be separated by the present design. (i) *Acquisition shift*: BRACS slides were digitized under a different scanner and staining protocol than TCGA and TIGER, producing a color and texture distribution the Stage 3 classifier never saw; we applied no stain normalization or color augmentation beyond the augmentations listed in Section 2.7, so the models had no explicit mechanism for absorbing this shift. (ii) *Annotation-protocol shift*: BRACS is annotated as regions of interest spanning a lesion spectrum, and collapsing that spectrum onto the binary tumor/non-tumor axis used here places borderline categories on one side of a boundary that the TCGA- and TIGER-derived labels draw elsewhere; part of the measured degradation is therefore label harmonization rather than model failure. (iii) *Prevalence and sample-size shift*: the BRACS test split is small (271 patches, 22 patients) and its class balance differs from the pooled test partition, so AUC is estimated with materially less precision than in the internal evaluation.

#### 3.9.3 Scope of the claim

This experiment establishes that both pipelines retain discriminative signal on an institution absent from every training stage; it does not establish institution-independent generalization. Note also that the AdCo backbone was not re-pre-trained for this experiment—it remains a CPTAC-BRCA model—so the comparison isolates the effect of the labeled pipeline rather than of the pre-training corpus. We return to the implications of the single-institution design in Section 4.7.

## 4 Discussion

### 4.1 Pseudo-label generator quality as the dominant performance driver

Across both strategies, the quality of the pseudo-label generator was the primary determinant of downstream semi-supervised classification performance, consistently outweighing the choice of SSL architecture. In Strategy 1, the AUC spread within a fixed PL model group reached at most 0.09 across SSL architectures, while the best AUC per PL model group ranged from 0.7985 to 0.8466. This pattern held in Strategy 2, where the within-group variance narrowed further to 0.03, but the generator-driven ordering was preserved. The evidence is unambiguous: investing in a stronger pseudo-label generator yields greater returns than searching over downstream architectures.

### 4.2 What the retention rates reveal

The confidence-filtered retention rates (Table 5) were remarkably close between strategies, with Strategy 2 exceeding Strategy 1 by only 2–3 percentage points per architecture. This finding has two important implications. First, the AdCo backbone—trained entirely without labels on 347,884 unlabeled CPTAC-BRCA patches—achieved the same degree of decisiveness at θ = 0.85 as a supervised model explicitly trained to classify tumor from non-tumor tissue. This demonstrates that adversarial contrastive pre-training on domain-specific unlabeled data produces a feature space at least as linearly separable as that of a label-scarce supervised model, through instance discrimination alone rather than direct class supervision.

Second, the near-identical retention rates confirm that the downstream performance gap between the two strategies (Tables 3, 6) was not attributable to differences in pseudo-labeled set size. Both strategies provided Stage 3 with a comparable quantity of unlabeled patches; the differences in AUC floor and cross-architecture variance therefore reflect differences in pseudo-label *accuracy* rather than volume. The AdCo backbone assigned high-confidence predictions to patches that were genuinely unambiguous, while supervised generators—particularly ResNet-18, whose low-AUC pseudo-labels drove the worst Strategy 1 results—produced equally confident but less accurate assignments, consistent with confirmation bias in label-scarce models (Lee, 2013).

The retention rate is nonetheless a threshold statistic and is insensitive to the *margin* of confident predictions: a prediction of 0.86 and a prediction of 0.98 count identically. The AUC results provide the more diagnostic comparison; future work should examine the full predicted probability distribution across both strategies to more precisely characterize differences in calibration and confidence margin.

### 4.3 AdCo pre-training: benefits and limitations

Strategy 2 produced a consistently higher AUC floor (0.8274 vs. 0.7462 in Strategy 1) and substantially narrower within-group variance (0.03 vs. 0.09), consistent with adversarial contrastive pre-training yielding more reliable pseudo-labels than supervised generation under limited annotation. The largest absolute gains accrued to architectures that suffered most from weak pseudo-labels in Strategy 1: ConvNeXt-Small as an SSL model improved by +0.0329 AUC when switching from supervised to AdCo pseudo-labels, compared to +0.0135 for ResNet-18, which was less sensitive to generator quality.

However, the gains were moderate rather than dramatic, which reflects two genuine limitations of the AdCo pipeline as deployed here. First, there is a domain gap between pre-training data (CPTAC-BRCA) and the labeled set (TIGER, BRACS, and TCGA): differences in scanner hardware, staining protocol, and tissue preparation mean the AdCo backbone was exposed to a distribution that only partially overlaps with the data on which pseudo-labels are evaluated. The close retention rates confirm this gap did not critically impair the linear probe, but it constrained the magnitude of the improvement. The cross-institution experiment (Section 3.9) is consistent with this reading: on the entirely held-out BRACS institution, both strategies declined, with Strategy 2 recording the higher AUC (0.78 vs. 0.74). The confidence intervals overlap, so this comparison is indicative rather than conclusive and we do not treat it as evidence of superior transfer. The ablations (Section 3.8) show the same ordering across every confidence-threshold and loss-weight setting tested, indicating the observed difference is not contingent on a favorable hyperparameter choice, though its magnitude remains small throughout. Second, the AdCo objective optimizes for *instance* discrimination, not class-level separability: the backbone learned to distinguish individual patches from one another, and the tumor/non-tumor axis emerged as a downstream consequence rather than a direct training target. A class-conditioned contrastive objective that explicitly groups tumor and non-tumor patches during pre-training would more directly align the representation with the classification task.

### 4.4 Failure modes

Aggregate AUC conceals the structure of the errors both strategies make. Four failure modes are visible in the results reported above, and we describe them explicitly because each has a different remedy.

#### 4.4.1 Specificity-limited errors on non-tumor tissue

The confusion matrices in Figure 5A show that errors are strongly asymmetric: at the default threshold both strategies recover roughly 89% of tumor patches while misclassifying 32.9% (Strategy 1) and 26.6% (Strategy 2) of non-tumor patches as tumor. The dominant failure is therefore over-calling tumor on benign tissue, not missing tumor. AdCo pre-training reduces this failure by 6.3 percentage points at matched sensitivity, which is the largest single-metric difference between the two strategies anywhere in our results and is consistent with a pre-trained representation that separates histological texture more cleanly than a label-scarce supervised generator.

#### 4.4.2 Over-confidence, and its coupling to pseudo-label noise

The calibration curves (Figure 5D) lie below the diagonal throughout, so both models systematically overstate their own certainty. This is not only a deployment concern: the same scores drive the confidence filter that produces the pseudo-labels. A threshold of θ = 0.85 therefore does *not* retain a set that is 85% precise, and the pseudo-labeled corpus entering Stage 3 is noisier than the nominal threshold implies. The θ ablation (Table 9) shows why simply raising the threshold does not fix this: at θ = 0.95 retention collapses to 0.48 and 0.41 and AUC falls in both strategies, so the noise is traded against a loss of coverage rather than eliminated. Calibrating the generator before filtering—rather than filtering harder—is the appropriate remedy, and we did not attempt it here.

#### 4.4.3 Failure of the weak-generator regime

Strategy 1's worst combination reaches only 0.7462 AUC, and no downstream architecture recovers from it: a ResNet-18 pseudo-label generator trained on scarce labels produces confident but inaccurate assignments, which the Stage 3 model then reproduces. This is the confirmation-bias failure characteristic of self-training (Lee, 2013), and it is the specific failure that AdCo pre-training mitigates—the floor rises to 0.8274—rather than the peak-accuracy case, where the two strategies are indistinguishable.

#### 4.4.4 Failure modes the present design cannot characterize

The test partition draws 98.7% of its patients from TCGA, so per-institution error structure outside TCGA is estimated from very few patients; the TIGER test split in particular contains 162 tumor against 6 non-tumor patches, which is too imbalanced to support a stable threshold-independent error analysis. We therefore cannot report whether the failure modes above are shared across institutions or are TCGA-specific, and we do not claim that they are shared. Patch-level evaluation also leaves slide-level failure uncharacterized: a model may be accurate on the majority of patches while erring systematically on the small-area regions that determine a slide-level diagnosis, and the present design cannot detect this. Both gaps require a more balanced multi-institution test partition than the available data provided.

### 4.5 Cross-architecture transferability

A consistent practical finding across both strategies was that high-quality pseudo-labels transferred robustly to any downstream architecture. In Strategy 2, the best-performing PL backbone (ConvNeXt-Small) produced an AUC range of only 0.8388–0.8601 across the three SSL architectures—a spread of 0.0213—meaning that a practitioner could choose ResNet-18, DenseNet-121, or ConvNeXt-Small as the Stage 3 model without meaningful loss of performance. This architectural independence has direct practical value: once a strong pseudo-label generator is established, the downstream classifier can be chosen based on deployment constraints such as inference latency or memory budget rather than classification performance.

### 4.6 Practical implications

The results establish a clear design principle for semi-supervised WSI classification under label scarcity: the highest-leverage decision is the choice of pseudo-label generator. In both strategies, a single strong generator bootstrapped all three downstream architectures with comparable performance, while a weak generator (ResNet-18 pseudo-labels in Strategy 1, AUC floor of 0.7462) was not rescued by using a more powerful SSL architecture. This ordering was consistent across all 18 architecture combinations evaluated.

For practitioners deploying semi-supervised histopathology pipelines, these results suggest prioritizing pre-training data quality and generator architecture over SSL classifier selection. The benefit we can claim for AdCo pre-training is one of *robustness* rather than peak accuracy: no matched head-to-head AUC difference reached significance under the patient-clustered bootstrap (Section 3.6), but the performance floor rose by +0.0812 AUC and the cross-architecture spread halved, consistently and in the same direction across all nine paired combinations. The practical implication is that AdCo pre-training reduces the risk of a poor outcome from an unlucky architecture or generator choice, at no annotation cost, even when the unlabeled corpus originates from a different institution than the labeled set—not that it reliably raises the best achievable accuracy.

### 4.7 Limitations and scope

Three limitations bound the scope of the claims made here.

#### 4.7.1 External validation rests on a single institution

The cross-institution experiment (Section 3.9) is a leave-one-dataset-out evaluation on BRACS, not a multi-center study. One held-out institution is a single draw from the space of possible domain shifts, and a 0.08–0.11 AUC degradation measured on 22 patients constrains how precisely that shift can be characterized. We report the direction of the effect—both strategies transfer with substantial loss, Strategy 2 losing less—without claiming that the ordering would hold at another site. A full leave-one-institution-out protocol over four or more sources, with patient-level aggregation and a stain-normalization ablation to separate acquisition shift from biological and annotation differences, is the evaluation this question requires and the first target for follow-up work.

#### 4.7.2 Error analysis is constrained by the composition of the test partition

Section 4.4 characterizes the error structure of both strategies, but 98.7% of test patients come from TCGA, so that characterization is effectively single-institution. Whether the over-calling of benign tissue we observe is a general property of these pipelines or a TCGA-specific one cannot be resolved with the available data.

#### 4.7.3 Validation is retrospective and at patch level

All evaluation here is retrospective, on curated public cohorts, at the level of individual 512 × 512 patches. Prospective clinical validation—on consecutively accrued cases, with slide-level or case-level endpoints, and with measurement of the effect on pathologist reading—lies outside the scope of this study, which is a methodological comparison of two pseudo-labeling strategies. Two findings reported above are nonetheless prerequisites for any such study and would need to be resolved first: the systematic over-confidence documented in Section 3.7, which *post-hoc* recalibration would address, and the gap between patch-level and slide-level performance, which the present design does not measure. We make no claim of clinical readiness.

## 5 Conclusion

We proposed and compared two semi-supervised frameworks for breast cancer patch classification under limited annotation. Strategy 1 trained a supervised model on the available labeled data to generate confidence-filtered pseudo-labels, then trained a separate, independently ImageNet-initialized classifier on the combined labeled and pseudo-labeled data. Strategy 2 replaced the supervised pseudo-label generator with a backbone pre-trained via Adversarial Contrastive Learning (AdCo) on 347,884 unlabeled CPTAC-BRCA patches, using a frozen linear probe to generate pseudo-labels before the same semi-supervised fine-tuning stage.

Across all 18 architecture combinations evaluated, pseudo-label generator quality was the dominant driver of downstream performance, outweighing the choice of SSL classifier architecture. Strategy 1 achieved a best AUC of 0.8466 (ConvNeXt-Small → ResNet-18), approaching the fully supervised ceiling of 0.8627 while requiring substantially fewer ground-truth annotations. Strategy 2 raised the AUC floor from 0.7462 to 0.8274 and reduced within-group variance from 0.09 to 0.03, indicating that AdCo pre-training on domain-specific unlabeled data yields more consistent pseudo-labels than supervised generation under label scarcity. We note explicitly that no matched per-architecture AUC difference between the two strategies was statistically significant under patient-clustered bootstrapping; the evidence for Strategy 2 rests on the floor, spread, and the consistent direction of all nine paired differences rather than on any single head-to-head comparison. The AdCo linear probe achieved AUC = 0.84 on the held-out labeled partition using a frozen backbone and a single linear layer, demonstrating that adversarial contrastive pre-training learns genuinely transferable histological representations without any annotation.

The close pseudo-label retention rates between strategies (2–3 percentage points) established that the Strategy 2 improvement was driven by higher pseudo-label *accuracy* rather than volume, consistent with the AdCo backbone learning a more reliable representation of histological structure through instance discrimination rather than class-shortcut supervision.

These conclusions carry two bounds. Cross-institution validation rests on a single held-out institution of 22 patients, in which both strategies lost 0.08–0.11 AUC relative to within-institution testing, so deployed performance should be expected to fall well below the internal figures. And both strategies fail in the same characteristic way—over-calling tumor on benign tissue while remaining systematically over-confident—which propagates into the confidence-filtered pseudo-labels themselves. All evaluation is retrospective and at patch level; we make no claim of clinical readiness.

Within those bounds, the findings suggest a practical design principle for semi-supervised WSI classification: prioritize the pseudo-label generator over the downstream architecture. Future work should investigate class-conditioned contrastive objectives that more directly align pre-training with the classification task, adaptive confidence thresholding strategies, calibration of the pseudo-label generator prior to confidence filtering, and extension of the framework to slide-level classification via multiple instance learning.

## Data Availability

The original contributions presented in the study are included in the article/supplementary material, further inquiries can be directed to the corresponding author.
